# Possible mechanism of metabolic and drug resistance with L-asparaginase therapy in childhood leukaemia

**DOI:** 10.3389/fonc.2023.1070069

**Published:** 2023-02-01

**Authors:** Ruizhi Zhou, Tianqi Liang, Tianwen Li, Junbin Huang, Chun Chen

**Affiliations:** Department of Pediatrics, the Seventh Affiliated Hospital of Sun Yat-Sen University, Shenzhen, Guangdong, China

**Keywords:** L-asparaginase, asparagine synthetase, metabolic, drug resistance, childhood leukaemia

## Abstract

L-asparaginase, which hydrolyzes asparagine into aspartic acid and ammonia, is frequently used to treat acute lymphoblastic leukaemia in children. When combined with other chemotherapy drugs, the event-free survival rate is 90%. Due to immunogenicity and drug resistance, however, not all patients benefit from it, restricting the use of L-asparaginase therapy in other haematological cancers. To solve the problem of immunogenicity, several L-ASNase variants have emerged, such as *Erwinia*-ASNase and PEG-ASNase. However, even when *Erwinia*-ASNase is used as a substitute for *E. coli*-ASNase or PEG-ASNase, allergic reactions occur in 3%-33% of patients. All of these factors contributed to the development of novel L-ASNases. Additionally, L-ASNase resistance mechanisms, such as the methylation status of ASNS promoters and activation of autophagy, have further emphasized the importance of personalized treatment for paediatric haematological neoplasms. In this review, we discussed the metabolic effects of L-ASNase, mechanisms of drug resistance, applications in non-ALL leukaemia, and the development of novel L-ASNase.

## Introduction

L-asparaginase (L-ASNase), an enzyme that hydrolyzes asparagine, is one of the most successful drugs for metabolic targeting to date and one of the most important chemotherapeutic drugs in standardized regimens for childhood ALL. L-ASNase is essential for improving the complete remission rate and long-term survival in children with ALL. In the moderate/low-risk group mainly according to the treatment response of 15-19 days and the level of minimal residual disease in 29-45days, event-free survival and overall survival rates can reach 90% when combined with other chemotherapeutic drugs (1–3). L-ASNase (L-ASNase) has been shown to have anticancer activity that depends on its ability to hydrolyse asparagine since it was discovered in guinea pig serum in 1953 (4–7). In 1966, Dolowy et al. first reported complete remission in a case of refractory childhood acute lymphoblastic leukaemia (ALL) treated with guinea pig-derived L-ASNase (8). In 1970, Clarkson et al. first reported the treatment of ALL with purified *E. coli*-derived L-ASNase (*E. coli*-ASNase) and the induction of remission (9). In the following decades, L-ASNase was widely used in the treatment of ALL. Currently, the clinically used L-ASNases include *E. coli*-ASNase, *Erwinia*-ASNase, and PEG-ASNase, among which PEG-ASNase has the longest half-life and lowest immunogenicity (5, 10). Nevertheless, allergic reactions to L-ASNase still occur in 30%-70% of patients, which limits its efficacy (11). To provide new insights into using L-ASNase in treating paediatric leukaemia, we discussed the metabolic effects of L-ASNase, mechanisms of drug resistance, applications in non-ALL leukaemia, and the development of novel L-ASNase in this review.

## Metabolic effects of L-ASNase on leukaemic cells

Tumour cells have different metabolic patterns compared to normal cells. This metabolic pattern is manifested by increased glycolysis, glucose uptake, and uptake and catabolism of amino acids (12–14). Metabolic reprogramming allows tumour cells to show resilience in hypoxic and nutrient-deficient environments. At the same time, however, such metabolic alterations also make tumour cells exhibit specific vulnerabilities, such as an increase in certain specific metabolic demands (15). The increased metabolic demands determine the importance of glucose and amino acids in tumour metabolism. Unlike normal cells, amino acids that are not essential to normal cells may be essential to tumour cells because tumour cells usually lose the ability to synthesize these amino acids *de novo*, enabling the amino acid deprivation therapy.

**Figure 1:**
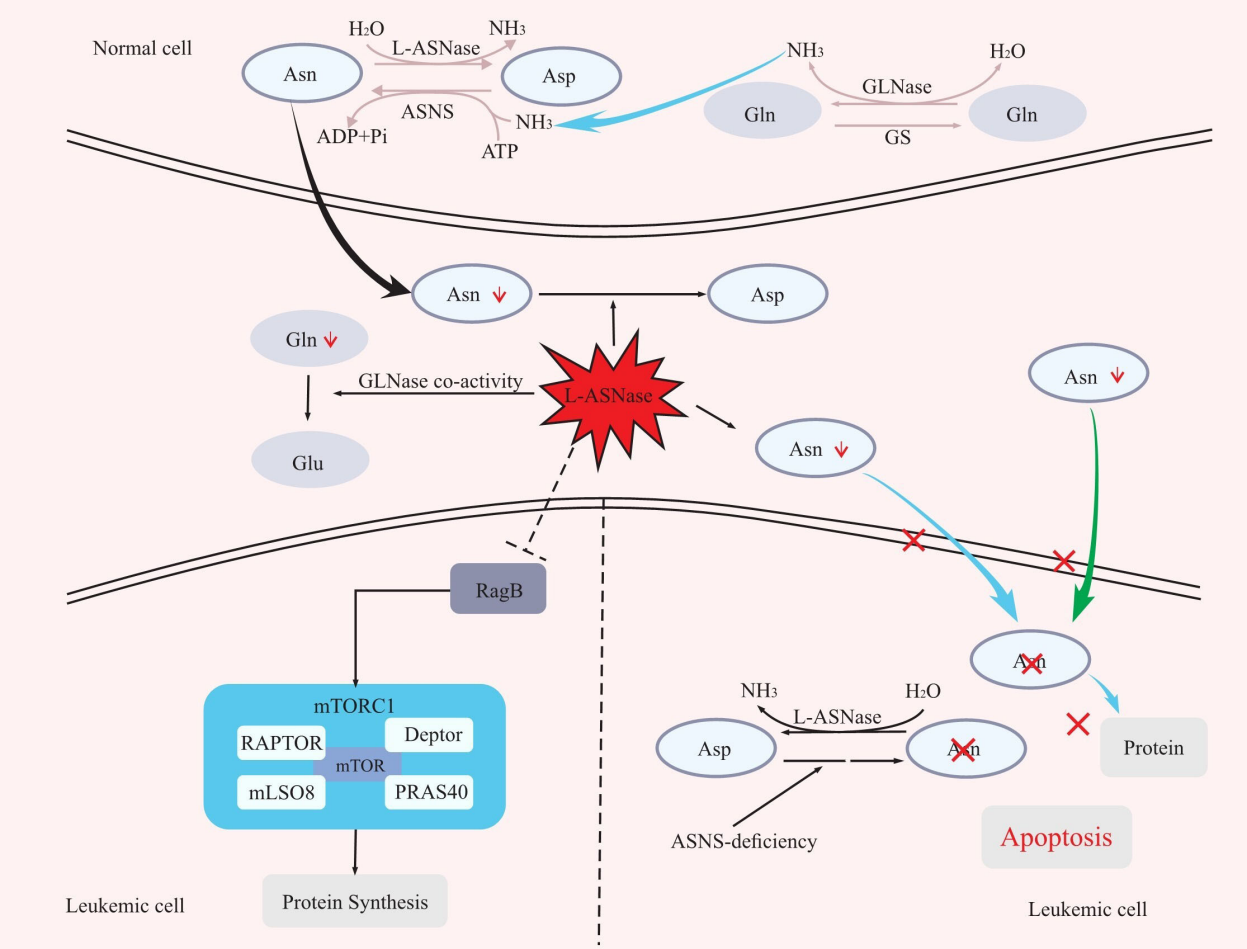
The mechanism of L-ASNase. L-ASNase depletes Asn, and GLNase coactivity hydrolyzes Gln, which further reduces Asn, leading to apoptosis of leukaemia cells. L-ASNase can inhibit RagB-mTORC1 and thus inhibit protein synthesis. Asp, aspartic acid; Asn, asparagine; Gln, glutamine; Glu, glutamic acid; ASNS, asparagine synthetase; L-ASNase, L-asparaginase; GS, glutamine synthetase; GLNase, glutaminase.

Asparagine is a nonessential amino acid involved in protein synthesis for normal cells, which can be obtained from food or produced by the combination of aspartate acid with ammonia catalysed by asparagine synthase (ASNS) (16, 17).Different from normal cells, due to the lack of ASNS, leukaemia cells frequently fail to synthesize asparagine and therefore must rely on the host to supply asparagine for their protein synthesis requirements. By catabolizing asparagine in serum, L-ASNase can expose leukaemia cells to an asparagine-deficient environment, and thus affecting protein synthesis in leukaemic cells and leading to their growth inhibition or death (5, 16–20). In addition, Hermanova et al. further demonstrated the molecular mechanism by which L-ASNase inhibits protein synthesis in leukaemic cells (21). The mammalian target of rapamycin protein complex 1 (mTORC1) plays a central role in the amino acid response. RagA/RagB switches from a GDP-bound state to a GTP-bound state as amino acid levels rise, which activates mTORC1, and in turn stimulates a series of downstream reactions, including protein synthesis (22). However, it was recently discovered that RagB-expressing cells can still activate mTORC1 even in an amino acid-deficient environment (23). By treating wild-type RagB cells and RagB-mutant cells (in a permanent GTP-bound state) separately with L-ASNase and assaying the levels of the mTORC1 downstream molecule p-S6 protein, Hermanova et al. found that wild-type RagB cells had significantly lower p-S6 protein levels, while RagB-mutant cells did not show significant changes in p-S6 protein levels. That is, L-ASNase can inhibit protein synthesis by inhibiting RagB-mTORC1 (21) (**Figure 1**).

L-ASNase also has glutaminase (GLNase) activity that can hydrolyze glutamine. In the presence of ASNS, glutamine can act as an amino donor to facilitate the production of aspartic acid into asparagine. Therefore, the hydrolysis of glutamine by L-ASNase also contributes to the reduction of asparagine levels, improving the efficacy (16, 19, 24) (**Figure 1**). However, whether the anti-leukaemic effect of L-ASNase depends on GLNase activity is controversial (25–32). First, Offman and Parmentier et al. demonstrated that the killing effect of L-ASNase on leukaemic cells was reliant on GLNase activity and that the cytotoxicity of L-ASNase on leukaemic cells increased with increasing GLNase activity (25, 29). Chan et al. also demonstrated in a recent study that L-ASNase with GLNase activity was more cytotoxic to leukaemic cells and could better prolong the survival of mice in an ASNS-negative SUP- B15 xenograft model (32). In contrast, a previous study showed that L-ASNase without GLNase activity could achieve the same level of antitumour effects as wild-type L-ASNase in ASNS-negative leukaemia cell lines (30). Nguyen et al. also demonstrated that L-ASNase mutants with low GLNase activity had the same level of antitumour activity as L-ASNase mutants with high GLNase activity in an ASNS-negative SUP-B15 leukaemia cell xenograft model (31). However, the L-ASNase in their research is not completely devoid of GLNase activity. Therefore, we believe that GLNase activity of L-ASNase is required for the killing effect of L-ASNase in ASNS-negative tumour cells, but at what level of GLNase activity needs to be maintained is a question that require confirmation through more experiments.

In addition to protein synthesis and amino acid metabolism, Hermanova et al. found that L-ASNase can also affect the energy metabolism of leukaemic cells, including increased fatty acid oxidation and inhibition of glycolysis. They suggested that the inhibition of mTORC1 by L-ASNase was responsible for inducing fatty acid oxidation. Moreover, they found that fatty acid oxidation inhibitors and L-ASNase can act synergistically to kill cells (21). Therefore, the combination regimen of fatty acid oxidation inhibitors with L-ASNase may provide a brand-new option for the treatment of ALL. Furthermore, Takahashi et al. demonstrated in their study that L-ASNase can inhibit glycolysis in leukaemic cells (18), but the precise molecular mechanism of this is unknown. In general, L-ASNase can affect the energy metabolism of leukaemia cells, which in turn may hurt the efficacy of L-ASNase. Clarifying the specific mechanism will provide new selections to ALL treatment, and more research on combined treatment regimens targeting energy metabolism such as fatty acid oxidation will bring new hope to ALL treatment.

## Mechanism of drug resistance

### Asparagine synthase

Studies on the mechanism of L-ASNase resistance have been widely conducted. Numerous studies have found elevated expression of ASNS in L-ASNase-resistant tumour cells. It has been confirmed that L-ASNase-resistant cells express higher levels of ASNS than L-ASNase-sensitive cells (33–36). Scherf and Holleman et al. reported that L-ASNase-sensitive cells express lower levels of ASNS mRNA *in vitro* (37, 38). In contrast, however, the results of Fine et al. did not find a correlation between the expression level of ASNS mRNA and sensitivity to L-ASNase (39). In B-lineage lymphocytic leukaemia cells carrying the TEL-AML 1 translocation, Stams et al. also obtained the same results as Fine et al. (40). Even in other studies, higher expression levels of ASNS mRNA were found in L-ASNase-sensitive TEL-AML1-positive cells compared with TEL-AML1-negative cells that were resistant to L-ASNase (41), but they did not further elucidate the relationship between TEL-AML1 fusion genes and ASNS gene expression. In addition, Su et al. stated that high ASNS expression did associate with resistance to L-ASNase. But they suggested that it should be the ASNS protein, rather than the mRNA, to be tested as indicators of L-ASNase resistance as there was no significant correlation between the levels of ASNS mRNA and ASNS protein (42). In any case, these studies illustrate the point that ASNS expression confers L-ASNase resistance in leukaemic cells.

Other studies have revealed that the methylation status of the *ASNS* promoter region can affect the transcription of *ASNS* and thus affect the sensitivity of L-ASNase. *ASNS* is part of the amino acid response pathway that is activated by amino acid deficiency (43, 44). When asparagine is deprived, tumour cells can respond *via* the GCN2-ATF4 pathway. ATF4 binds to the *ASNS* promoter in a hypomethylated state and induces its expression (45). Jiang et al. found that the hypermethylated state of the *ASNS* promoter restricted the binding of the transcription factor ATF4 upon amino acid depletion, and thereby inhibiting *ASNS* expression (46). Overall, amino acid deficiency-induced *ASNS* expression requires both GCN2 activation and hypomethylation of the *ASNS* promoter region, which enable ATF4 binding to drive *ASNS* expression. A cohort study by Akahane et al. further confirmed that the hypomethylation status of the *ASNS* promoter region is associated with L-ASNase resistance. Their analysis of 75 Japanese children with T-ALL revealed an intermediate (33.3% < methylation <66.7%) or low (<33.3%) methylation status of the *ASNS* promoter region in 92% of refractory/relapsed cases (47). In addition, Touzart et al. found ASNS to be expressed at low levels in TLX1^+^ T-ALL cells (high *ASNS* methylation levels). TLX1^+^ T-ALL was more sensitive to L-ASNase than the TLX^-^CCRF-CEM cell line (low *ASNS* methylation level) (48). Recently, the important role of amino acid stress response genes in L-ASNase sensitivity was further confirmed by Ferguson et al., who identified a novel L-ASNase resistance gene, *SLC7A11*, whose high expression leads to L-ASNase sensitivity in cancer cells (49). In conclusion, these studies all suggested that the hypomethylation status of the *ASNS* promoter region contributes to the expression of ASNS induced by L-ASNase treatment, and thus conferring L-ASNase resistance to leukaemic cells. However, there are limited cohort studies to refer to at present, therefore, additional larger cohort studies are needed to further confirm the possibility of *ASNS* promoter region methylation as a predictor of treatment response.

Meanwhile, the factors affecting the methylation status of the ASNS promoter region have been reported. Worton et al. reported that L-ASNase induces *ASNS* promoter demethylation, which confers drug resistance to leukaemic cells (50). However, the mechanism by which L-ASNase induces demethylation has not been further confirmed. The study by Akahane et al. focused on the significance of *SPI1* fusion in the methylation status of *ASNS*. In their cohort study, all seven *SPI1* fusion cases had an *ASNS* promoter hypomethylation status, and the *ASNS* gene expression levels were significantly higher than those of *SPI1* fusion-negative cases (47). This suggests that genetic modifications may play an important role in the methylation status of the *ASNS* promoter region. Yet, it is critical to confirm the relationship between poor prognosis-associated fusion genes and *ASNS* gene methylation status and the molecular mechanism of L-ASNase-induced demethylation, providing information for treatment option and improving the prognosis for ALL patients.

### Energy metabolism and autophagy

Several recent studies showed that L-ASNase resistance is related to phosphatase and tensin homologue (PTEN) deficiency and phosphatidylinositol-3 kinase (PI3K)/Akt/mTOR signalling pathway. PTEN is a major negative regulator of the PI3K/Akt/mTOR signalling pathway. Deletion of PTEN can occur in 20% of children with T-ALL and plays an important role in the development and prognosis of T-ALL in children (51–53). Hlozkova et al. proposed that the metabolic pattern of leukaemic cells is associated with L-ASNase resistance after investigating the effect of L-ASNase treatment on the extensive metabolic reprogramming of leukaemic cells. They found that cells with a high glycolytic response are resistant to L-ASNase (54). They subsequently confirmed the relationship between glycolytic levels and L-ASNase sensitivity by investigating the effects of PTEN deficiency on the metabolism of leukaemic cells and changes in L-ASNase sensitivity. Furthermore, a recent study by Hlozkova et al. found that, compared to PTEN wild-type cells, PTEN-deficient T-ALL cells have a higher glycolytic function and overactivated Akt, and these changes made T-ALL cells resistant to L-ASNase. Meanwhile, the resistance of PTEN-deficient cells to L-ASNase could be improved by inhibiting Akt signalling (53). These results suggest that Akt inhibitors may contribute to the treatment of T-ALL patients with PTEN mutations, but further experiments are still needed for verification.

Amino acid deprivation has been shown to induce the activation of autophagy, which is considered a self-protective mechanism in tumour cells (55–57). Hermanova et al. showed that L-ASNase can induce the activation of protective autophagy in leukaemia cells by inhibiting mTORC1 (21). As in previous studies (58), they suggested that autophagy could counteract nutrient imbalance by recycling amino acids, thus resisting the cytotoxicity of L-ASNase (21). Takahashi et al. also reported that L-ASNase treatment reduced glycolysis in leukaemia cells while causing mitochondrial damage and activating autophagy. However, they concluded that the function of L-ASNase-induced autophagy was to eliminate mitochondrial damage and thus reducing ROS production rather than amino acid recycling. Notably, in this study, they demonstrated that by inhibiting autophagy, the cytotoxicity of L-ASNase could be enhanced and such synergistic effect works through the ROS-p53 positive feedback loop (18). In addition, Takahashi et al. and Polak et al. further confirmed that autophagy inhibitors and L-ASNase have synergistic anti-leukaemic effects (59, 60). The activation of autophagy is one of the mechanisms leading to L-ASNase resistance, but the role of autophagy in this mechanism still needs to be further refined. The abovementioned studies suggest that timely detection of autophagy activation during L-ASNase treatment would be more helpful in the selection of treatment regimens, and the combination of L-ASNase with autophagy inhibitors may provide better clinical outcomes.

### Host factor

#### The role of the bone marrow haematopoietic microenvironment

The tumour microenvironment affects the cytotoxicity of L-ASNase (61). A study by Iwamoto et al. revealed the interaction between leukaemic cells and their surrounding microenvironment. The expression of ASNS is much higher in normal bone marrow mesenchymal stem cells (MSCs) than in leukaemic lymphoblastoid cells. *In vitro*, leukaemic cells can acquire resistance to L-ASNase by receiving asparagine from MSCs (62). Glutamine synthetase expression is increased in bone marrow adipocytes after induction of chemotherapy with L-ASNase, producing more glutamine and thus protecting leukaemic cells from L-ASNase (63). Future studies focusing revealing the molecular mechanism of the interaction between leukaemia cells and the haematopoietic microenvironment in the bone marrow will further elucidate the anti-leukaemic effect of L-ASNase and hence improving the L-ASNase therapy.

#### Neutralizing antibodies and silent inactivation

Due to its immunogenicity, an L-ASNase treatment can cause an immune response, which is associated with the production of neutralizing antibodies. Neutralizing antibodies can inactivate L-ASNase, and thereby reducing efficacy. The production of neutralizing antibodies in patients without clinical symptoms is known as silent inactivation, which is usually not clinically evident and thus difficult for early detection (11, 64, 65). Although it has been suggested that patients with allergic reactions to *E. coli*-ASNase and PEG-ASNase should be switched to *Erwinia*-ASNase (66), 3-33% of patients can develop an immune response against *Erwinia*-ASNase, resulting in neutralizing antibodies against L-ASNase and thus resistance to L-ASNase (67, 68).

#### Other pathways

It has been shown that leukaemic cells can acquire L-ASNase resistance through the OPRM1-cAMP-caspase pathway. Kang et al. identified the opioid receptor μ1(OPRM1) as a key factor for L-ASNase resistance in paediatric ALL using unbiased genome-wide RNAi. They analysed OPRM1 expression levels in primary leukaemic cells from five children with ALL in relation to L-ASNase sensitivity and found that cells with low levels of OPRM1 were more resistant to L-ASNase (69). In addition, Lee et al. identified the Huntington-associated protein 1 gene (*HAP1*) as an L-ASNase resistance gene, and by examining the relationship between HAP1 levels and L-ASNase sensitivity in the cells of six ALL patients, they found that the lower HAP1 level, the more resistant they were. Furthermore, they found that HAP1 deletion prevented Ca^2+^ release from the endoplasmic reticulum and downregulated the Calpain-1-Bid-caspase-3/12 pathway, conferring L-ASNase resistance in leukaemic cells (70). Additionally, a recent study demonstrated that if the Wnt pathway is blocked, cells may degrade proteins *via* GSK3-dependent protein ubiquitination and proteasome degradation pathways to synthesize asparagine to counteract the cytotoxicity of L-ASNase (71).

In conclusion, ASNS expression remains a pivotal factor in the resistance of L-ASNase in leukaemic cells, and ASNS expression is closely related to the methylation status of its promoter region. In addition, activation of autophagy, high glycolysis levels, or inhibition of apoptotic signalling pathways can all promote L-ASNase resistance. The gradual uncovering of L-ASNase resistance mechanisms further emphasizes the significance of individualized therapy and continues to provide new ideas for the further development of individualized combination therapy regimens.

## Application of L-ASNase in other childhood leukaemia

Although L-ASNase is currently used primarily for the treatment of ALL and some NK/T-cell lymphomas, there is growing evidence that L-ASNase can play a critical role in the treatment of other childhood leukaemias (26).

Dübbers et al. found that leukaemic cells from M1, M4, and M5 subtypes had negative ASNS staining among all FAB subtypes of AML and that AML-M5 had the lowest ASNS activity (72). This is in agreement with the results of Okada et al., who found L-ASNase to be effective against specific subtypes of AML (M1, M4, M5) *in vitro* (73). Additionally, according to Buaboonnam et al., patients with refractory/relapsed AML who received treatment with L-ASNase in combination with MTX had 1- and 2-year survival rates of 35.6% and 17.8%, respectively (74). Whether this regimen can be used as a treatment for patients with refractory/relapsed AML after intensive therapy still needs further study. More recently, Chen et al. reported that the combination of L-ASNase with MIT and Ara-C for AML could enhance the inhibition of tumour cell proliferation (75). It is the current belief that the toxic effect of L-ASNase on AML may be related to GLNase activity. Glutamine is a nutrient that AML cells require. L-ASNase can remove glutamine and thus inhibit the growth of AML cells. However, L-ASNase simultaneously promotes the production of glutamine synthetase, leading to L-ASNase resistance (76, 77). Thus, further research is still needed to clarify the role of the GLNase activity of L-ASNase. Furthermore, as in ALL, it has been shown that the bone marrow haematopoietic microenvironment protects AML cells. Cells in the bone marrow microenvironment can either release ASNS to counteract L-ASNase action or release lysosomal cysteine protease B (CTSB) to inactivate L-ASNase, which confers L-ASNase resistance (78)

The potential of L-ASNase in CML treatment has been uncovered. Song et al. found that L-ASNase inhibited growth and induced apoptosis in the human CML cell Lines K562 and KU812, among which the apoptosis induction of K562 cells by L-ASNase was dependent on caspase3 (79). This discovery makes it possible to use L-ASNase in the treatment of CML. Trinh et al. also demonstrated that L-ASNase could inhibit the growth of CML cells, and the combination of L-ASNase and imatinib can significantly induce CML cell death by downregulating antiapoptotic factors such as Bcl-2 and upregulating proapoptotic factors such as Bim, and thereby eradicating CML stem cells (80). A recent study by Konhauser et al. also demonstrated the synergistic effect of L-ASNase in combination with etoposide on killing K562 cells (81).

With the continuous development of studies on the metabolic and nonmetabolic effects of L-ASNase on paediatric leukaemia, studies on the therapeutic effects of L-ASNase on other non-ALL leukaemia are proliferating. These studies suggest that L-ASNase may provide a new option for the treatment of other paediatric leukaemias. These results are based on the enzymatic activity of L-ASNase, which depletes asparagine and glutamine in the blood and inhibits mTOR, which in turn affects protein synthesis and induces apoptosis. Meanwhile, these studies found that L-ASNase caused the activation of protective autophagy in tumour cells, so the combination of L-ASNase and autophagy inhibitors will benefit both ALL patients and non-ALL patients.

## Novel L-asparaginase

As mentioned above, L-ASNase is a xenogeneic protein agent that is highly immunogenic. Efficacy is compromised during L-ASNase treatment because of immunological or nonimmunological side effects. *Erwinia*-ASNase is often chosen as an alternative treatment for patients with *E.coli*-ASNase allergy (82), and PEG-ASNase has been introduced into the clinic for its longer half-life and lower immunogenicity. However, none of these variants can completely solve the problem. Neutralizing antibodies can still be produced and therefore inactivating L-ASNases (83, 84). To address these issues, several approaches have been used to develop novel L-ASNase preparations, such as reduced GLNase coactivity of L-ASNase, enzyme engineering modifications, and vector packaging.

Since most of the nonimmunological side effects of L-ASNase are attributed to GLNase activity, reducing the GLNase coactivity of L-ASNase may effectively ameliorate the side effects of L-ASNase. Consequently, L-ASNase variants with or without negligible GLNase activity were generated. Wolinella succinogenes-derived L-ASNase (*WOA*) was the first reported L-ASNase variant with low GLNase activity that did not suppress immune responses in mice (85–87). Reinert et al. showed no significant changes in glutamine in the liver and spleen of mice treated with the *WOA* variant compared to L-ASNase (88). Recent studies have also identified a guinea pig-derived humanized variant of L-ASNase that is completely devoid of GLNase activity. This variant has reduced immunogenicity while maintaining anti-leukaemic activity (89, 90).

Enzyme engineering has been widely employed to change the characteristics of L-ASNase in search of L-ASNase with low immunogenicity, a longer half-life, and lower GLNase activity. Since L-ASNase can be cleaved by CTSB and aspartate endopeptidase (78, 91), Offman et al. used site-directed mutagenesis to design an L-ASNase variant that is resistant to CTSB cleavage and has lower immunogenicity. They also designed a variant with low GLNase activity, N24A/R159S, which reduced the toxicity of L-ASNase (25). Furthermore, in a recent study, Maggi et al. designed an N24S mutant with improved protease resistance and thermostability in response to the instability and brief half-life of *E. coli*-ASNase (92).

In addition to the above methods, carrier packaging can also be used to reduce the immunogenicity of L-ASNase and make it more stable *in vivo*. Common carriers include erythrocytes, liposomes, nanocapsules, and microcapsules. The performance of these L-ASNases has also been demonstrated *in vivo* and *in vitro* (93–95). For instance, because it is encapsulated within cells, Eryaspase, a product that encapsulates *E. coli*-ASNase into erythrocytes, has a long half-life similar to that of erythrocytes and has low immunogenicity (96–98). Last year, Eryaspase was approved by the FDA for the treatment of ALL patients who are allergic to PEG-ASNase (99).

## Discussion

In summary, L-ASNase is still the cornerstone drug for the treatment of paediatric ALL. In addition to affecting the protein synthesis and amino acid metabolism of ALL cells, L-ASNase can affect energy metabolism. Also, changes in energy metabolism and autophagy in ALL cells may affect the efficacy of L-ASNase. The focus of current research on the mechanism of L-ASNase resistance is gradually shifting from the protein level to the gene expression regulation level. Meanwhile, there are studies that elucidate the relationship between leukaemia metabolic profiles and autophagy and L-ASNase resistance. Although the mechanism of L-ASNase resistance has not been fully elucidated to date, these studies suggested that the combination of fatty acid oxidation inhibitors or autophagy inhibitors and L-ASNase can provide better anti-leukaemic effects, which provide brand-new options for the future treatment of childhood leukaemia.

The immunogenicity of L-ASNase is a reason for its drug resistance. Using carrier packaging L-ASNase such as erythrocytes and nanocapsules can effectively reduce its immunogenicity and therefore L-ASNase can work better. The performance of these L-ASNases has also been demonstrated *in vivo*. Moreover, the necessity of GLNase activity for the anticancer effect of L-ASNase is still highly controversial. Although the development of L-ASNase variants with low GLNase activity continues, the necessity of GLNase activity and the level of GLNase activity that should be maintained for L-ASNase still needs to be further investigated. Moreover, some glutamine-dependent haematological tumours may not benefit from L-ASNase variants without GLNase activity.

Finally, addressing the above issues will not only help to solve the problem of ALL resistance to L-ASNase but also help to explain the potential application of L-ASNase in other tumours.

## Author contributions

TLia and TLi performed the collection and interpretations of all relevant literature. RZ wrote the manuscript. CC and JH critically read and revised the manuscript. All authors contributed to the article and approved the submitted version.
